# Col. George E. Waring

**Published:** 1898-11

**Authors:** 


### OBITUARY.

THE death of Col. George E. Waring, at New York, October 29, 1898, is to be rated as a distinct national loss. He contracted yellow fever at Havana while on special service for the government, hence sacrificed his life as distinctly as though he had fallen in the front rank before Santiago. He has performed such conspicuous service throughout the United States as a sanitary engineer, that his death will be felt by the medical profession as keenly as if one of its own members had been stricken down with the dread disease that Col. Waring was laboring to eradicate.

